# Genetic Variation of *G6PD* and *CYP2D6*: Clinical Implications on the Use of Primaquine for Elimination of *Plasmodium vivax*

**DOI:** 10.3389/fphar.2021.784909

**Published:** 2021-11-26

**Authors:** Alexandra G. A. Stewart, Peter A. Zimmerman, James S. McCarthy

**Affiliations:** ^1^ Infectious Diseases Unit, Western Health, Melbourne, VIC, Australia; ^2^ The Center for Global Health and Diseases, Case Western Reserve University, Cleveland, OH, United States; ^3^ Victorian Infectious Diseases Service, Royal Melbourne Hospital, Melbourne, VIC, Australia; ^4^ Peter Doherty Institute of Infection and Immunity, Melbourne, VIC, Australia

**Keywords:** Plasmodium vivax, primaquine, CYP2D6, G6PD (glucose-6-phosphate dehydrogenase), pharmacogenetics, elimination, mass drug administration (MDA)

## Abstract

Primaquine, an 8-aminoquinoline, is the only medication approved by the World Health Organization to treat the hypnozoite stage of *Plasmodium vivax* and *P. ovale* malaria. Relapse, triggered by activation of dormant hypnozoites in the liver, can occur weeks to years after primary infection, and provides the predominant source of transmission in endemic settings. Hence, primaquine is essential for individual treatment and *P. vivax* elimination efforts. However, primaquine use is limited by the risk of life-threatening acute hemolytic anemia in glucose-6-phosphate dehydrogenase (G6PD) deficient individuals. More recently, studies have demonstrated decreased efficacy of primaquine due to cytochrome P450 2D6 (*CYP2D6*) polymorphisms conferring an impaired metabolizer phenotype. Failure of standard primaquine therapy has occurred in individuals with decreased or absent CYP2D6 activity. Both G6PD and CYP2D6 are highly polymorphic genes, with considerable geographic and interethnic variability, adding complexity to primaquine use. Innovative strategies are required to overcome the dual challenge of G6PD deficiency and impaired primaquine metabolism. Further understanding of the pharmacogenetics of primaquine is key to utilizing its full potential. Accurate *CYP2D6* genotype-phenotype translation may optimize primaquine dosing strategies for impaired metabolizers and expand its use in a safe, efficacious manner. At an individual level the current challenges with G6PD diagnostics and CYP2D6 testing limit clinical implementation of pharmacogenetics. However, further characterisation of the overlap and spectrum of G6PD and CYP2D6 activity may optimize primaquine use at a population level and facilitate region-specific dosing strategies for mass drug administration. This precision public health approach merits further investigation for *P. vivax* elimination.

## Introduction

*Plasmodium vivax* and *P. ovale* are unique human malaria species in their ability to develop into hypnozoites, a liver stage that can remain dormant until relapse occurs weeks to years later (Krotoski 1985). Previously considered a benign disease there is now clear evidence that *P. vivax* can cause severe malaria (Baird 2013). Relapses can result in significant morbidity, and provide the predominant source for ongoing transmission in endemic settings, with up to 85% of *P. vivax* blood stage infections occurring due to reactivation of dormant hypnozoites (Ross et al., 2016; Commons et al., 2020). This poses a considerable challenge for global elimination efforts. Clearance of hypnozoites requires an 8-aminoquinoline (8AQ), such as primaquine (PQ) or tafenoquine (TQ), to achieve radical cure. However, use of 8AQ derivatives is limited by the risk of life-threatening acute haemolytic anemia (AHA) in glucose-6-phosphate dehydrogenase deficient (G6PDd) individuals. Additionally, because this risk is difficult to quantify in pregnancy, lactating women and infants, 8AQs are contraindicated in these groups. This safety concern has hampered widespread use, both at the individual level and as an elimination tool through mass drug administration (MDA).

Recently an additional issue with PQ efficacy has been identified, when multiple cases of *P. vivax* relapse were reported in patients treated with standard courses of PQ (Bennett et al., 2013, Ingram et al., 2014). The lack of PQ efficacy has been associated with cytochrome P450 2D6 (*CYP2D6*) polymorphisms conferring impaired metabolizer phenotypes of drug substrates of this hepatic detoxification enzyme (Baird et al., 2018b). There is significant geographic and interethnic variability in CYP2D6 metabolizer phenotypes, with high proportions of impaired metabolizers in *P. vivax* endemic areas, which may have considerable implications for the role of PQ for *P. vivax* elimination (Baird et al., 2018a).

Hence, despite 8AQs being in clinical use for more than 60 years, malaria-endemic countries remain unable to utilize their full potential. With no alternative hypnozoiticidal agents nearing licensure, innovative solutions are required to target the hypnozoite reservoir.

## Primaquine – Safety and Efficacy Issues

### Safety – Glucose-6-Phosphate Dehydrogenase Deficiency

**FIGURE 1:**
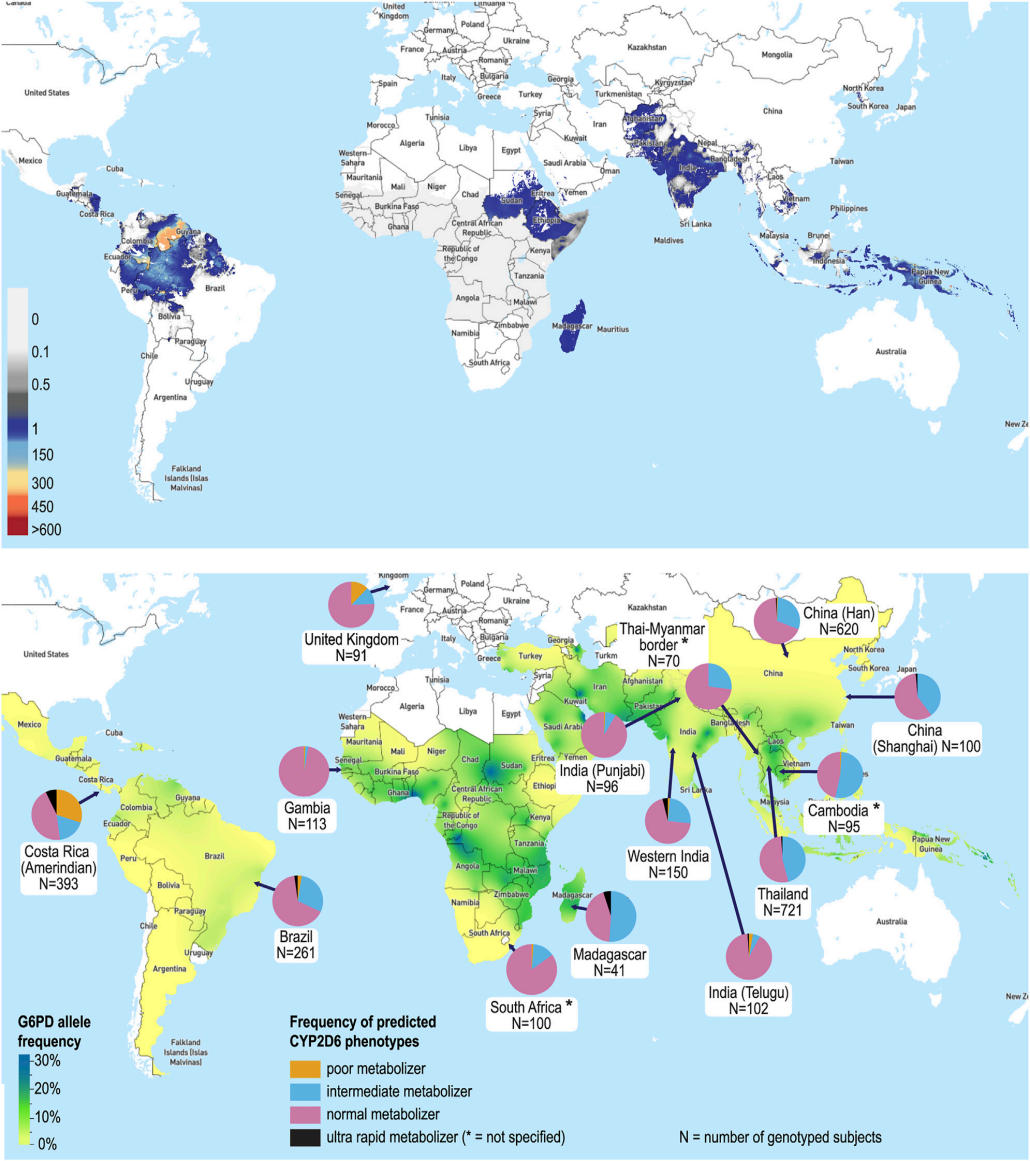
The global distribution of *Plasmodium vivax*, G6PDd and CYP2D6 metabolizer status. **(A)** Predicted incidence of *P. vivax* in 2017 [incidence in cases per 1,000 people per year are shown on a spectrum of white (zero incidence) to dark grey (1 case per 1,000) and then blue to red (>1 case per 1,000 to >600 cases per 1,000)] (Battle et al., 2019). **(B)** Map of the median predicted allele frequency of G6PDd (Howes et al., 2012), and CYP2D6 metabolizer status by country and/or ethnicity (selected sample represented) (Dodgen et al., 2016; Leitão et al., 2020; Spring et al., 2020; Koopmans et al., 2021; Mehlotra et al., 2021). Maps used with permission from the Malaria Atlas Project (https://malariaatlas.org).

Primaquine has been the mainstay of hypnozoiticidal therapy since it was first licenced as an anti-malarial in 1952. The description of AHA in G6PDd individuals taking PQ in 1956 was one of the earliest described pharmacogenetic associations (Dern et al., 1954; Alving et al., 1956). The X-linked defect of G6PDd has a highly polymorphic genotype, with 217 mutations identified (Gómez-Manzo et al., 2016). Enzyme activity varies depending on the severity of the variant and the gender of the patient (Luzzatto and Seneca 2014). In malaria-endemic countries the estimated frequency of deficiency alleles is 8% (Howes et al., 2012). However, there is considerable geographic and interethnic variability in G6PDd, with prevalence of G6PDd up to 32.5% in some regions **(** Figure 1 **)** (Howes et al., 2012). In *P. vivax* endemic countries 14.3% of the population are estimated to be ineligible for PQ based on G6PDd and contraindications of pregnancy, lactation and age <6 months (Baird et al., 2018a). Currently, WHO recommends G6PD testing prior to PQ administration; however in most malaria-endemic countries PQ is withheld due to inability to test G6PD activity (Recht et al., 2018; World Health Organization 2021). Regulatory authorities have recently approved single dose TQ for *P. vivax* radical cure. However, due its long terminal elimination half-life (12–16 days) and the risk of AHA, higher G6PD activity (70%) is required, significantly limiting its use (Lacerda et al., 2019; Chu and Hwang 2021). Thus, PQ remains the only hypnozoiticidal agent recommended by WHO for radical cure of *P. vivax* (World Health Organization 2021).

### Efficacy – CYP2D6 Polymorphisms

Primaquine is a pro-drug that requires metabolic transformation to metabolites active against hypnozoites. Primaquine’s mechanism of action is complex and still to be definitively defined. However, the hydroxylation pathway has been demonstrated in mouse and human studies to be CYP2D6 dependent (Pybus et al., 2012; Pybus et al., 2013; Potter et al., 2015; Popovici et al., 2021). Clinical evidence for CYP2D6 mediated metabolism, and its role in PQ efficacy comes from initial observations by Bennett et al., in 2013, where PQ treatment failures occurred in two subjects with *CYP2D6* genotypes conferring impaired metabolism (Bennett et al., 2013). Further evidence of clinical failures associated with impaired CYP2D6 activity has been demonstrated in studies from Papua New Guinea, Indonesia, Brazil and China (Ingram et al., 2014; Silvino et al., 2016; Baird et al., 2018b; Brasil et al., 2018; Silvino et al., 2020; Huang et al., 2021). Over 20% of the population in *P. vivax* endemic areas are estimated to carry *CYP2D6* alleles conferring impaired enzyme function, and are therefore at risk for PQ treatment failure (Baird et al., 2018a).

As outlined in the accompanying perspective piece by Olvany et al., the activity score (AS) metric, based on genotype, is used to predict the phenotype translation (poor (PM), intermediate (IM), normal (NM) and ultra-rapid metabolizers (UM)). Heterogeneity in the genotype-phenotype relationship has been observed, with significant inter- and intra-individual phenotypic variation (Gaedigk et al., 2018). In addition to single nucleotide polymorphisms, small insertions and deletions, copy number variations and the non-functional CYP2D7 pseudogene make it challenging to accurately assign phenotypes using genotyping approaches (Del Tredici et al., 2018; Nofziger et al., 2020). Additional genetic modifiers are hypothesized to play a role in phenotypic variability, and greater understanding of these modifiers is required to accurately determine metabolizer status (Gaedigk et al., 2018).

Regional variations in CYP2D6 enzyme activity are well characterized **(** Figure 1 **)**. While populations of European heritage have the highest frequency of PMs, common reduced function variants (e.g., *10, *17 and *41) result in a higher frequency of IMs in East Asian, African and Middle Eastern populations (Sistonen et al., 2007; LLerena et al., 2014). The allele conferring reduced function, CYP2D6*10, predominates in Southeast Asia (frequency up to 40%), the region with the highest *P. vivax* burden*,* with 1.4 billion people at risk (Gething et al., 2012; LLerena et al., 2014). Population admixture adds further complexity, as extrapolation by ethnicity is hampered by differences in biogeographical ancestry, unique allele combinations and frequency patterns (Suarez-Kurtz et al., 2014; Nofziger et al., 2020). From a clinical perspective pharmacogenetic knowledge of interethnic variability and admixture can influence choice of drugs for national formularies and dosing guidelines (Roederer and McLeod 2010). For example, in Brazil warfarin dosing algorithms based on CYP2C9 and VKORC1 genes were unreliable in regions with different population admixture (Botton et al., 2011; Suarez-Kurtz 2011).

Drug-drug and food-drug interactions can also influence CYP2D6 metabolic activity, with concomitant CYP2D6 inhibitors causing phenoconversion (Sasaki et al., 2017; Gaedigk et al., 2018; Nofziger et al., 2020). Similarly, physiologic and environmental factors may affect the CYP2D6 phenotype and AS (Gaedigk et al., 2018). Further characterization of these interactions, the need for adjustment to phenotype prediction or potential dosing algorithms will be required to ensure accurate application of PQ pharmacogenetics in clinical practice.

## Clinical Implications

### G6PDd and CYP2D6 Pharmacogenetics in Clinical Practice

Rapid progress in pharmacogenetic research has led to increased recognition of clinically actionable gene-drug pairs, with more than 50 drugs identified by the international Clinical Pharmacogenetics Implementation Consortium (CPIC) and the Dutch Pharmacogenetics Working Group (DPWG), and development of pharmacogenetic labelling (Swen et al., 2011; Yoon et al., 2020). However, there has been slow translation of pharmacogenetic testing and guided prescribing into clinical practice.

This is particularly true for malaria–despite the known association of G6PDd with PQ for over 60 years qualitative point-of-care (POC) G6PD diagnostics have only recently become available, and use remains limited in many areas (Thriemer et al., 2017). The first generation of these tests report enzyme activity >30% as “normal” and hence are not suitable for determining eligibility for TQ (LaRue et al., 2014). Additionally, qualitative tests cannot identify female heterozygotes with intermediate activity, and they therefore remain at risk of clinically significant hemolysis (Chu et al., 2017). Promisingly, quantitative POC G6PD diagnostics have recently been developed (e.g. SD Biosensor Standard™ G6PD), allowing identification of individuals with intermediate activity (Alam et al., 2018; Pal et al., 2019). While these represent significant progress, issues of accessibility, usability and cost remain (Thriemer et al., 2017).

Pharmacogenetic testing for *CYP2D6* diplotypes has the potential to play a significant role in patient management prior to use of PQ, particularly for IM where alternative dosing strategies may be required. However, limitations for clinical CYP2D6 testing include laboratory expertise required, prolonged turnaround time, cost, low number of alleles included in commercial testing (particularly for those in less well studied populations), accuracy issues due to short-read sequencing and incomplete *CYP2D6* genotype databases (Haga 2016; Hippman and Nislow 2019). These limitations preclude the use of pharmacogenetic testing in *P. vivax* endemic areas. In practice POC testing would be required for clinical use. However, due to the complexity of the *CYP2D6* gene locus, this is not yet possible. Importantly, POC CYP2D6 testing would need to include common variations in regions where the test is deployed, given the geographic and ethnic variability in *CYP2D6* diplotypes (Haga 2016).

Implementation of clinical pharmacogenetic testing requires accurate prediction of phenotype and corresponding dosing guidelines. Prior discrepancies in the categorization of AS and metabolizer status between CPIC and DPWG guidelines have now been resolved, with recent standardization of CYP2D6 genotype-phenotype translation (Caudle et al., 2020). Uptake of this consensus translation system by clinical laboratories and therapeutic guidelines will ensure consistent pharmacogenetic implementation. Activity scores can be used in high-resource settings to make therapeutic recommendations, such as for codeine (Crews et al., 2014). However, further refinement of the CYP2D6 genotype-phenotype relationship is required to make such AS recommendations for PQ (Gaedigk et al., 2018).

### Primaquine Dosing

Optimizing PQ dosing will be essential in G6PDd and impaired PQ metabolizers, as the total dose of PQ administered, influences efficacy for radical cure, while AHA occurs in a dose-dependent manner, with decreased dosing frequency used as a strategy to mitigate this risk in populations with milder variants (John et al., 2012). In individuals with G6PDd of the African A^−^ variant weekly dosing of PQ over 8 weeks (0.75 mg base/kg/week) has been shown to be a safe alternative to daily dosing for 14 days (0.25–0.5 mg base/kg/day), allowing for controlled hemolysis (Alving et al., 1960). However, the safety of such PQ dosing depends on the G6PDd variant in question, with safety of this regimen less clear in patients with more severe G6PDd variants (e.g. Mediterranean, Mahidol, Viangchan). In Pakistan, no hematological adverse effects were noted in a G6PDd patient (likely Mediterranean variant) included in a randomized trial of weekly PQ (Leslie et al., 2008). However, in Cambodia, where the Viangchan variant predominates, significant drops in hemoglobin were noted with weekly dosing, including a patient that required blood transfusion (Kheng et al., 2015). Drug-drug interactions may have compounded hemolysis in this patient (Kheng et al., 2015), highlighting the clinical complexity, including the need to consider concomitant medications, both from a safety and efficacy perspective.

*P. vivax* relapses after PQ therapy are most commonly associated with *CYP2D6* diplotypes with an AS < 0.5; however relapses have also occurred in individuals with diplotypes with an AS = 1.5, a NM phenotype (Baird et al., 2018b). Therefore, a greater understanding of the role of genetic variation on PQ metabolism is required, and no definitive AS threshold for radical cure has been established. Potential dosing strategies, as outlined by Olvany et al., will need to balance an increased PQ dose for efficacy with the potential risk of AHA. In a recent randomized control trial high dose short-course PQ (1 mg base/kg/day for 7 days) was non-inferior and well tolerated in G6PD normal individuals (Taylor et al., 2019), highlighting an approach worth testing in clinical trials to assess greater PQ efficacy in IM.

## Public Health Implications

### Mass Drug Administration for *P. vivax* Elimination

Given the significant challenges integrating G6PDd and CYP2D6 phenotypes into individual case management, a population approach, utilizing pharmacogenetics in MDA merits consideration. Historically, PQ use in MDA has been an effective strategy for *P. vivax* control in eradication settings (Newby et al., 2015). In the majority of settings PQ was administered in the absence of G6PD testing (where known G6PDd prevalence varied between 1 and 39%) (Newby et al., 2015). However, close monitoring was undertaken and adverse effects were rare (Newby et al., 2015). In analysis of daily PQ use in these MDA programs the incidence of significant hemolysis was estimated at 1.8 cases per million exposed (Recht et al., 2014). The MDA strategy led to suppression of transmission in Papua New Guinea, China, Afghanistan, Azerbaijan, Tajikistan and Democratic People’s Republic of Korea, and sustained interruption of transmission on Aneityum island, Vanuatu (Kaneko et al., 2000; Hsiang et al., 2013; Kondrashin et al., 2014; Newby et al., 2015). Currently the WHO does not recommend MDA for *P. vivax* (World Health Organization 2020), in large part due to the recommendation for G6PD testing prior to PQ administration. Some experts believe that PQ for radical cure can be administered in certain populations without G6PD testing, depending on the balance of population-risk of hemolysis versus the benefits of radical cure (Thriemer et al., 2017). In appropriately selected regions PQ for radical cure is administered without G6PD testing. In southern Papua (G6PDd prevalence 3%) the beneficial effects of PQ, such as lower risk of *P. vivax* related severe anemia, hospital admission or representation, outweighed the risks (Thriemer et al., 2020). However, in the Brazilian Amazon two deaths secondary to PQ-induced AHA have been reported (Lacerda et al., 2012). In this region G6PDd prevalence is also 3% (predominantly the mild A^−^ variant) (Santana et al., 2009). This highlights the risk of rare but life-threatening adverse effects when PQ administration is based on population data.

Without the ability to test all individuals for G6PDd the acceptable risk-benefit balance in PQ MDA remains unresolved. Although treatment of *P. vivax* infection confers direct benefit to the individual, when used in MDA, some participants may not be hypnozoite carriers, and therefore at risk of harm with no possible clinical benefit (Jamrozik et al., 2015). Further, if population coverage is poor then risks of adverse events secondary to PQ may outweigh the overall benefits of an MDA program aiming for elimination (Cheah and White 2016).

Achieving success with MDA depends on the therapeutic efficacy of the drug administered and ensuring 80–90% population coverage (Newby et al., 2015; Tanner et al., 2015). With expanding knowledge of the effect of *CYP2D6* polymorphisms on PQ efficacy this must be factored into MDA planning. Baird et al. have estimated that 38.8% of the population living in *P. vivax* endemic areas would be excluded from receiving standard PQ regimens based on G6PDd and impaired PQ metabolism (Baird et al., 2018a). Hence, with current PQ dosing regimens it may not be possible to reach the population threshold for interruption of transmission. Utilizing population knowledge of G6PDd and *CYP2D6* genotypes may facilitate dosing strategies that reduce the proportion of individuals currently deemed “ineligible” for radical cure and allow coverage thresholds for MDA to be reached.

### The Role of Pharmacogenomics in MDA - Challenges and Potential Solutions

Population-scale sequencing projects, such as the 1,000 Genome Project, provide a global overview of genetic diversity and interethnic variability (Abecasis et al., 2012). However, genomic databases continue to significantly under-represent developing countries and ethnically diverse populations (Jarvis et al., 2019; Sivadas and Scaria 2019). National and regional population screening programs are gathering pace (e.g. H3 Africa, African Genome Variation Project, SEAPharm) and will contribute to closing this gap (Gurdasani et al., 2015; Mulder et al., 2018; Chumnumwat et al., 2019). These projects may lead to higher resolution mapping of G6PDd and *CYP2D6* polymorphisms. The unique overlay and spectrum of G6PDd and impaired PQ metabolism will influence population-dosing algorithms for safe and efficacious use of PQ in specific regions. One significant challenge is the highly polymorphic nature of both the CYP2D6 and G6PD genes. The combined complexity may make integrating pharmacogenetic knowledge at a population level challenging.

Modeling suggests that ascending dose regimens in mild-moderate G6PDd may be effective and well tolerated, with optimal regimens allowing for slow hemolysis and minimal drops in hemoglobin without the need for G6PD testing (Watson et al., 2017). Further modeling to incorporate increased dosing for impaired PQ metabolizers into ascending dose regimens may facilitate strategies to ensure both safety and efficacy in MDA. Projected population coverage, taking into account regional pharmacogenetic-guided dosing regimens, will inform regional feasibility of MDA, and whether an acceptable risk-benefit threshold is met. Although these are complex challenges to navigate there is the potential for reducing *P. vivax* burden through region-specific MDA strategies, aligned with the WHO “High burden to high impact approach”, a country led, targeted strategy, as opposed to the current dogma of one-size fits all (World Health Organization 2019).

## Future Directions

Currently the use of pharmacogenetics for *P. vivax* radical cure is beyond reach due to insufficient understanding of the role of CYP2D6 in PQ metabolism and efficacy. A number of key knowledge gaps **(** Table 1 **)** must be addressed; including the clinical and public health implications of *CYP2D6* and *G6PD* pharmacogenetics, to ensure translation into clinical practice is pragmatic for *P. vivax* endemic areas.

**TABLE 1:** Key knowledge gaps in CYP2D6 and G6PDd pharmacogenetics for PQ use in *P. vivax* radical cure.

Knowledge gaps
Pharmacogenetics
• Role of *CYP2D6* polymorphisms on PQ metabolism and efficacy
• Role of other genetic modifiers on CYP2D6 activity and PQ metabolism
• Understanding of the role of external confounders on CYP2D6 activity and PQ metabolism
• Accurate *CYP2D6* genotype-phenotype translation for PQ metabolism
• Determination of phenotypic risk in different G6PDd genotypes
• Investigation of whether both PQ-induced hemolytic toxicity and efficacy is mediated by a common metabolic pathway
Clinical
• Understanding of the relationship between PQ dose and efficacy in individuals with impaired CYP2D6 phenotypes
• Understanding of the relationship between PQ dosing requirements for efficacy and PQ dosing for safety
• Modeling and clinical trials to determine safe and efficacious dosing strategies based on AS
Public Health
• High resolution mapping of both CYP2D6 metabolizer and G6PDd status
• Impact of population admixture on CYP2D6 metabolizer status
• Analysis of risk-benefit balance of blind PQ administration in *P. vivax* eliminating countries
• Analysis of the feasibility and cost-effectiveness of quantitative POC G6PD diagnostics in MDA

Firstly, will tailored dosing strategies be effective? Modeling is the initial step, but ultimately clinical trials are required to assess dosing regimens based on population G6PDd and PQ metabolizer status. If evidence supports tailored dosing strategies to account for population G6PDd, will national malaria control programs support MDA of PQ without testing? Region-specific dosing will not completely mitigate the risk of PQ toxicity; the level of acceptable risk-benefit balance and ethical issues surrounding use of PQ in MDA require further debate. In populations where the risk outweighs the benefit, quantitative G6PD testing prior to PQ administration may facilitate higher dosing to ensure safety and efficacy.

Altered PQ dosing regimens may be more complex, with potential for poor adherence, risk of incorrect administration and interactions with concomitant drugs or foods that increase the risk of AHA or decrease the efficacy of PQ due to CYP2D6 inhibition. Will this complexity be too high for MDA operational feasibility? If blind administration is approved rigorous pharmacovigilance will be required.

Will it be possible to reach the 80–90% MDA coverage required for successful elimination of *P. vivax*? In addition to the well-described operational challenges in rolling out MDA, from a pharmacogenetic perspective, population admixture will need to be taken into account. Will it be possible to achieve safe and efficacious population dosing guided by pharmacogenetic data?

Population pharmacogenetics integrated in national public health policy to guide safe and efficacious PQ dosing for radical cure has the potential to enable MDA for *P. vivax* elimination, and build towards individualized case management as progress is made with POC pharmacogenetic testing. This is a challenging prospect, and one that ultimately may not be feasible. However, it deserves further investigation given the limited drugs available for *P. vivax* radical cure and the underutilization of PQ to date.

## Data Availability

The original contributions presented in the study are included in the article/Supplementary Material, further inquiries can be directed to the corresponding author.
